# MICAL1 facilitates pancreatic cancer proliferation, migration, and invasion by activating WNT/β-catenin pathway

**DOI:** 10.1186/s12967-022-03749-1

**Published:** 2022-11-12

**Authors:** Kun Cai, Lu Deng, Dijie Zheng, Lin Li, Zhiwei He, Chao Yu

**Affiliations:** 1 Department of Hepatic-Biliary-Pancreatic Surgery, The Affiliated Hospital of Guizhou Medical University, No.28 Guiyi Street, Yunyan District, Guiyang, 550001 Guizhou China; 2 College of Clinical Medicine, Guizhou Medical University, Guiyang, China; 3 College of Basic Medicine, Guizhou Medical University, Guiyang, China; 4 Guizhou Provincial Institute of Hepatobiliary, Pancreatic and Splenic Diseases, Guiyang, China; 5 Key Laboratory of Liver, Gallbladder, Pancreas and Spleen, Guizhou Medical University, Guiyang, China; 6 Guizhou Provincial Staff Hospital, Guiyang, China; 7 Department of Hepatobiliary Surgery, Shenzhen Key Laboratory, Shenzhen University General Hospital, Xueyuan AVE 1098, Nanshan District, Shenzhen, 518055 Guangdong China; 8 Shenzhen University Clinical Medical Academy Center, Shenzhen University, Shenzhen, China

**Keywords:** Pancreatic cancer, MICAL1, WNT pathway, TBC1D1, FZD7

## Abstract

**Background:**

MICAL1 is involved in the malignant processes of several types of cancer; however, the role of MICAL1 in pancreatic cancer (PC) has not been well-characterized. This study aimed to investigate the expression and function of MICAL1 in PC.

**Methods:**

RT-qPCR and immunohistochemistry were used to detect MICAL1 expression in PC and adjacent nontumor tissues. Cell Counting Kit-8, EdU, clone formation, wound healing, and Transwell assays as well as animal models were used to investigate the effects of overexpression or inhibition of MICAL1 expression on the proliferation, invasion, and metastasis of PC cells. RNA-seq was used to explore the main pathway underlying the functions of MICAL1. Proteomics, mass spectrometry, and co-immunoprecipitation assays were used to investigate the interaction of proteins with MICAL1. Rescue experiments were conducted to validate these findings.

**Results:**

Both MICAL1 mRNA and protein levels were upregulated in PC tissues compared with matched adjacent nontumor tissues. The expression level of MICAL1 was associated with the proliferative and metastatic status of PC. Repression of MICAL1 significantly inhibited PC cell growth, migration, and invasion in vitro and in vivo. RNA sequencing analysis indicated that MICAL1 was closely correlated with the WNT pathway. Overexpression of MICAL1 (1) promoted the phosphorylation of TBC1D1 at the Ser660 site, (2) facilitated the distribution of FZD7 on the cytomembrane, (3) inhibited the degradation of FZD7 in the lysosome, and (4) activated the WNT pathway.

**Conclusions:**

MICAL1 was upregulated in PC and involved in stimulating the progression of PC cells by activating the WNT/β-catenin signaling pathway. Therefore, MICAL1 is a potential therapeutic target for PC.

**Supplementary Information:**

The online version contains supplementary material available at 10.1186/s12967-022-03749-1.

## Background

Pancreatic cancer (PC) is a fatal disease with almost as many deaths as new cases per year [1]. With no effective screening test and nonspecific symptoms at an early stage, only 10–20% of patients with PC have a chance to receive curative surgery [2]. With the development of adjuvant therapy, the 5-year overall survival rate of PC has increased from 3% in the 1970s to nearly 9% in 2020 [1, 3]. This modest improvement in survival rate is much less than that recorded for many other tumor types [4]. Furthermore, PC mortality is projected to continue to rise over the next few decades, with over 800 000 deaths expected by 2040 based on the assumption that mortality rates will be stable from 2020 to 2040. Thus PC represents a major medical challenge [5, 6].

Molecules interacting with casL (MICALs) are a family of actin-regulatory oxidation–reduction (redox) enzymes that directly bind and disassemble actin filaments (F-actin) [7]. Three vertebrate MICAL genes (*MICAL1*, *MICAL2*, and *MICAL3*) and one *Drosophila* MICAL gene have been identified and are collectively called the MICALs [8].

MICAL1 was first discovered in 2002 and it is increasingly being implicated in various pathologies, including different cancers [9–12], diabetic nephropathy [13], susceptibility to infection [14], epilepsy [15], and cardiac protection [16]. Recent research shows that MICAL1 disruption attenuated breast cancer tumour growth and migration in vivo and MICAL1 related Rac1 activation facilitates hypoxia-induced gastric cancer cell migration [12, 17]. These findings indicate that pharmacological MICAL1 inhibition could have therapeutic benefits for corresponding cancer patients. However, to date, the role of MICAL1 in the pathogenesis and progression of PC has rarely been reported, whether MICAL1 could be a potential therapeutic target for pancreatic cancer is still unclear.

Increasing evidence indicates activation of the WNT/β-catenin pathway in almost all aspects of human tumor initiation and progression, including stemness, growth, survival, drug resistance, angiogenesis, immune evasion, and metastasis [18]. When WNT glycoproteins (Wnts) interact with seven transmembrane receptors-Frizzled (FZD), the WNT signaling pathway is activated and AXIN and APC are recruited to the cytomembrane. This results in the stabilization and nuclear localization of β-catenin [19, 20]. In the nucleus, β-catenin binds to the TCF/LEF, thus recruiting coactivators CBP to induce WNT target gene transcription and ultimately promoting the occurrence and development of tumors. The combination between Wnts and FZD receptors plays a vital role in cancer progression [21].

In this study, we demonstrated that MICAL1 was amplified in PC tissues and that it played an auxo-action in PC progression by activating the WNT/β-catenin pathway. Thus, MICAL1 is a potential therapeutic target for improving the clinical prognosis of patients with pancreatic cancer.

## Methods

### Bioinformatics analysis

Gene Expression Profiling Interactive Analysis (GEPIA, http://gepia.cancer-pku.cn/index.html) was used to verify the transcriptional levels of MICAL1 in 171 normal pancreatic samples from the GTEx coupled with TCGA database and 179 PC samples from TCGA. GSE15471 (36 tumor samples and 36 nontumor samples) and GSE28735 (45 tumor samples and 45 nontumor samples) were downloaded from The Gene Expression Omnibus (GEO, https://www.ncbi.nlm.nih.gov/geo/).

### Sample collection

Ninety-two pairs of tumor and adjacent normal tissue were collected from patients with PC who underwent surgery at the Department of Hepatobiliary Surgery, Affiliated Hospital of Guizhou Medical University (Guiyang, China). All patients signed a written notification of agreement, and the collection and usage of clinical samples were approved by the Ethics Committee of Guizhou Medical University.

### Cell culture

The telomerase-immortalized human pancreatic duct-derived cells (HPNE) and six lines of PC cells (AsPc-1, BXPC-3, CFPAC-1, PANC-1, MIA-PaCa2, and SW1990) were obtained from the American Type Culture Collection. HPNE, AsPc-1, and BXPC-3 cells were cultured in RPMI-1640 medium (Gibco) containing 10% fetal bovine serum (FBS, Gibco), whereas Capan-2, CFPAC-1, PANC-1, MIA-PaCa2, and SW1990 were cultured in DMEM containing 10% FBS. All cells were authenticated by STR profiling, routinely tested and confirmed to be mycoplasma-free, and maintained under standard conditions at 37 °C and 5% CO_2_.

### Cell transfection and lentiviral infection

MICAL1-overexpressing lentivirus, MICAL1-silencing lentivirus, and their corresponding control lentivirus were obtained from Genechem (Shanghai, China). To construct stable-expressing cell lines, cells were cultured with puromycin (1 μM, Invitrogen) for 2 weeks after lentiviral infection for 48 h. Small interfering RNAs (siRNAs) for TBC1D1 and FZD7 were obtained from RiboBio (Guangzhou, China). Target sequences of relevant genes used are listed in Additional file 5: Table S1. A pseudophosphorylated mutant of TBC1D1 on Ser660 (TBC1D1-S660D) plasmids, a nonphosphorylatable mutant of TBC1D1 on Ser660 (TBC1D1-S660A) plasmids, TBC1D1-overexpressing plasmids, and their corresponding control plasmids were obtained from Genechem. Transient transfection of plasmid or siRNA was performed using Lipo3000 (Invitrogen) following the manufacturer’s guidelines.

### RT-qPCR

Total RNA was isolated from the cells and tissues using the TRIzol reagent (Takara). RNA was reverse-transcribed to cDNA through the PrimeScript™ RT reagent Kit (Takara). Amplification was performed on a CFX96 Touch Real-Time Fluorescence Quantitative PCR Instrument (Bio-Rad Laboratories) using TaKaRa TB Green^™^ Premix Ex Taq^™^ II (Takara). GAPDH was used as a loading control. The 2^−ΔΔCt^ method was used to quantify targeted mRNA expression. Three independent replicates were analyzed. The primer sequences used in this study are listed in Additional file 5: Table S2.

### Cell proliferation assays

Cell Counting Kit-8 (CCK-8) and 5-ethynyl-2′-deoxyuridine (EdU) assays were used to evaluate the vitality of cell proliferation. For the CCK-8 assay, 3 × 10^3^ cells were cultured in 96-well plates, with six duplicate wells per group. The CCK-8 reagent (MedChemExpress) was added into each well according to the manufacturer’s guidelines at the indicated time intervals after the conglutination of cells. The absorbance of each well was measured at 450 nm after conventional culture for 2 h. For the EdU assay, 4 × 10^4^ cells were cultured in 12-well plates. Then EdU reagent (Guangzhou RiboBio Co., Ltd) was added into each well according to the manufacturer’s guidelines. The EdU-positive nuclei ratio of six random microscope views (20 × 10 magnification) per treatment was used and three independent replicates were analyzed.

### Colony formation

For the colony formation assay, 1 × 10^3^ cells were plated in a 6-well plate. After 2 weeks of culture, the cells were fixed with 4% paraformaldehyde (PFA) before they were stained with 0.5% crystal violet solution. Three independent replicates were analyzed.

### Transwell assays

The 8 μm migration Transwell chambers (Corning) were used for the Transwell assays. For the cell migration assay, 5 × 10^4^ cells and 200 µL of FBS-free medium were added to the upper chambers whereas 700 µL of medium containing 10% FBS was added to the lower chambers. After 24 h of culture, the upper chambers were fixed with 4% PFA and stained with 0.5% crystal violet solution. After removing the non-migrated cells on the upper surface of the upper chamber, the migrated cells were photographed using an optical microscope. Six random microscope views (20 × 10 magnification) per treatment were observed and three independent replicates were analyzed. For cell invasion assays, the upper surface of the chambers was coated with 25 µL Matrigel (Sigma); all other steps were the same as those of the cell migration assay described above. Three independent replicates were analyzed.

### Western blot analysis

For western blot analysis, 50 µg of cell lysis protein was separated by SDS–PAGE, electro-transferred to polyvinylidene fluoride membrane (Millipore), and blocked in 5% bovine serum albumin (BSA). Membranes were immunoblotted with the corresponding antibodies for 10 h at 4 °C, followed by the appropriate HRP-conjugated affinipure goat anti-mouse/rabbit antibodies for 2 h at 20 °C. Immunoreactive bands were visualized using extremely sensitive ECL reagent (Boster Biological Technology Co. Ltd.) with a chemiluminescence imaging system (Tanon). GAPDH was used as a loading control. Antibodys and dilution ratios of relevant genes used are listed in Additional file 5: Table S3.

### Immunohistochemistry

The tumor and adjacent normal tissue samples were fixed, embedded, sectioned, and deparaffinized. The sections were blocked using 3% H_2_O_2_ and 5% BSA, after which they were incubated with a corresponding antibody for 10 h at 4 °C. After incubation with secondary antibody and staining with diaminobenzidine. IHC score was calculated as the sum of the score for staining intensity multiplied the score for percentage of positive stained cells blindly by two individuals. The staining intensity was scored in four levels (absent = 0, weak = 1, moderate = 2, and strong positive = 3). The percentage of positive staining cells was scored in 6 levels (< 1% = 0, 1–25% = 1, 26–50% = 3, 51–75% = 4, 76–100% = 5). Scores less than the median was considered low expression and scores greater than or equal to the median were considered high expression.

### Immunofluorescence

Cells were fixed with 4% paraformaldehyde (PFA), permeabilized with 0.3% Triton, blocked in 5% BSA, and stained with the corresponding antibodies for 10 h at 4 °C. After they were incubated with fluorescence-labeled secondary antibodies, the cell nuclei were counterstained with DAPI. The cells were viewed and photographed using a fluorescence microscope (Leica).

### Immunoprecipitation assays

The co-transfected cells with plasmids or untreated cells were harvested after 48 h and lysed using NP-40 lysis buffer (Beyotime, Shanghai, China). After centrifugation, the supernatants were incubated with antibodies for 10 h at 4 °C, followed by adding Protein A + G beads for 3 h of incubation at 4 °C. After washing five times in the lysis buffer, the samples were blended with SDS–PAGE loading buffer, degenerated at 100 °C for 10 min, and examined by western blotting. IgG was used as a negative control.

### Top/Fop-Flash reporter assay

TOP/FOP-Flash reporter plasmids (Beyotime, Shanghai, China) were used according to the manufacturer’s instructions. Co-transfected with 50 ng Top flash or Fop flash expression plasmids together with other plasmids or siRNA after 5 × 10^3^ cells were plated in 96-well plates for 12 h. Then the dual luciferase assay Kit (Beyotime, Shanghai, China) was used to detect the luciferase activity 48 h after transfection and the TOP/FOP ratio was calculated to determine the Wnt/β-catenin pathway’s activity. Three independent replicates were analyzed.

### In vivo* experiments*

Protocols of animal experiments were approved by the Ethics Committee of Guizhou Medical University (Guiyang, China). BALB/cA-nu mice (6 weeks old, HFK Bio-Technology Co., Ltd, Beijing, China) were randomly assigned to each group. To evaluate the proliferation capacity in vivo, 2 × 10^6^ cells resuspended in 100 µL of PBS were inoculated to the right flank of mice. Tumor volumes were measured every 5 days. The tumor tissues were isolated from euthanized mice 30 days after injection, weighted and used for immunohistochemistry. To evaluate the metastatic capacity in vivo, 1 × 10^6^ cell resuspended in 100 µL PBS was injected into the caudal vein of nude mice. Those mice were euthanized 60 days after injection and the metastatic foci in the lung were detected by HE staining.

### Statistical analyses

Experiments were replicated at least three times. Data were analyzed using SPSS 23.0 (IBM Corporation, Armonk, NY, USA) and presented as mean ± standard deviation (SD). If there is no additional description, differences between groups were analyzed using the two-tailed unpaired Student’s t-test. Survival was analyzed using the log-rank test. All the results with *P* < 0.05 were considered statistically significant, and were reported as: *, *P* < 0.05; **, *P* < 0.01; ns, *no significance*.

## Results

### MICAL1 was overexpressed in PC tissues and cells

**Fig. 1:**
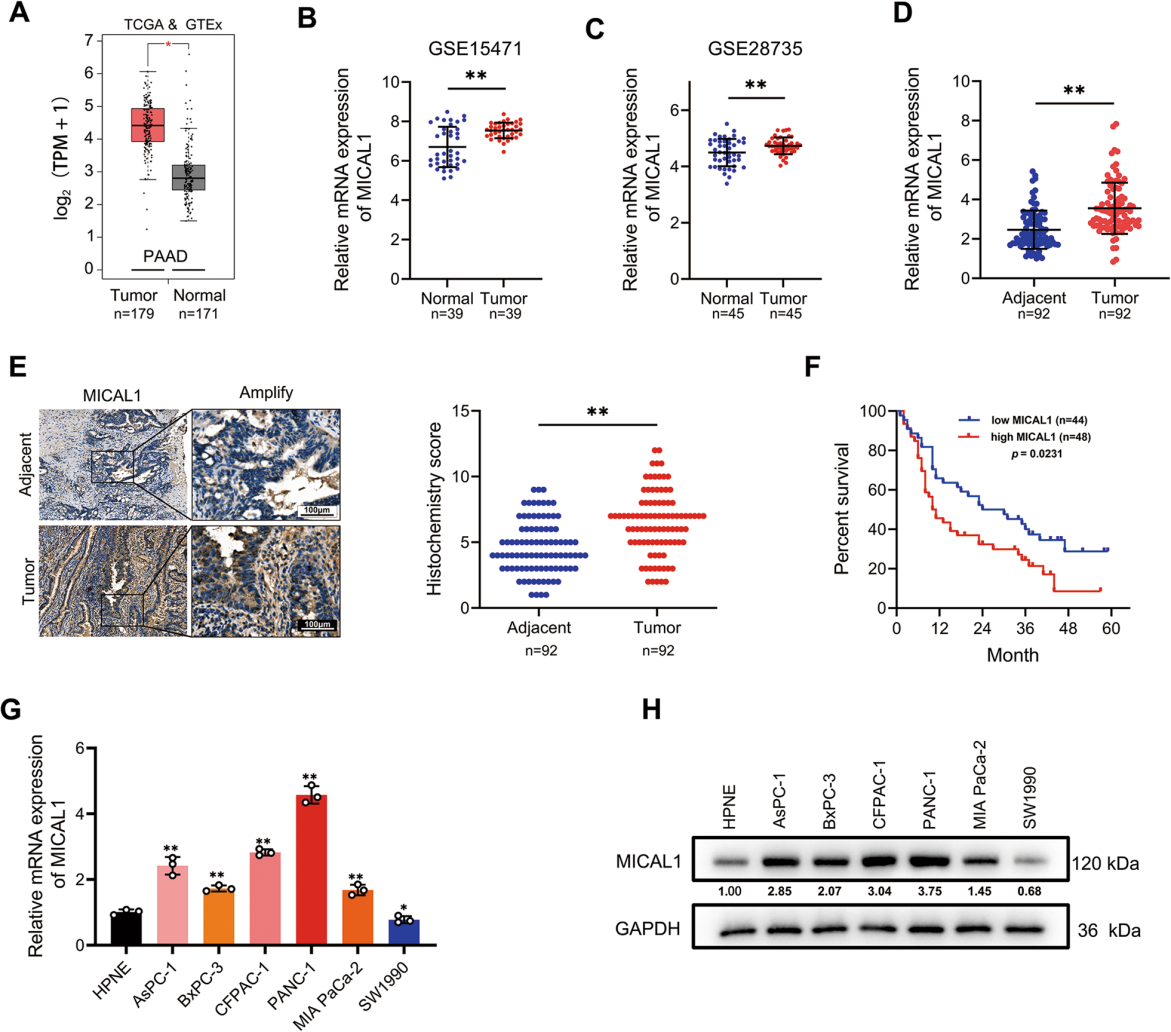
MICAL1 was overexpressed in PC tissues and cells. **A** MICAL1 mRNA expression in 179 PC tissues and 171 normal pancreatic tissues based on GTEx and TCGA databases. **B** MICAL1 mRNA expression in 39 normal pancreatic tissues and 39 PC tissues based on GSE15471. **C** MICAL1 mRNA expression in 45 normal pancreatic tissues and 45 PC tissues based on GSE28735 datasets. **D**, **E** RT-qPCR (**D**) and IHC (**E**) detected the expression of MICAL1 in 92 PC tissues and adjacent tissues. Scale bar, 100 μm. **F** Kaplan–Meier plots representing probabilities of overall survival in 92 PC patients according to the median histochemical score. Statistical analysis was conducted using the log-rank test. **G**, **H** MICAL1 mRNA (**G**) and protein (**H**) expressions in HPNE cells and described PC cells were tested**.** Except for **F**, statistical analysis was conducted using the two-sided unpaired Student’s t-test, *P < 0.05, **P < 0.01

To confirm the transcriptional levels of MICAL1, 171 normal pancreatic samples and 179 PC samples were analyzed by GEPIA. The results showed that MICAL1 expression in PC tissues was higher than that in normal pancreatic tissues (Fig. 1A). Results showed the same trend in the analysis of two GEO datasets, GSE15471 and GSE28735 (Fig. 1B and C).

To confirm this expression difference obtained from bioinformatics studies, we collected 92 matching pairs of pancreatic tumor and adjacent non-tumor tissues, measured mRNA expression by qRT-PCR, and detected protein expression by immunohistochemistry (IHC). The results indicated that both mRNA and protein levels of tumor tissues were higher than those of adjacent nontumor tissues (Fig. 1D and E). Moreover, analyses of the correlations between MICAL1 IHC scores and clinicopathological characteristics indicated that high MICAL1 expression was correlated with higher T and N classification (Table 1). Kaplan–Meier analysis also showed that clinical samples with high MICAL1 expression were associated with relatively poorer overall survival than those with low MICAL1 expression (Fig. 1F).

**Table 1:** The relationship between MICAL1 expression and clinical traits were analyzed using a chi-square test

Clinicopathologic feature	MICAL1		*P*
	High expression	Low expression	
All cases	48	44	
Age			
≤ 50	9	5	0.3245
> 50	39	39	
Gender			
Male	29	22	0.3153
Female	19	22	
T classification			
T1	21	30	***0.0185***
T2-4	27	14	
N classification			
N0	12	21	***0.0232***
N1/N2	36	23	
M classification			
M0	41	39	0.6469
M1	7	5	
AJCC stage			
I/II	33	34	0.3587
III/IV	15	10	

Bold values indicate statistically significant, *P* values less than 0.05

Simultaneously, we tested MICAL1 expression in HPNE cells and 6 PC cell lines. Except for SW1990, the mRNA (Fig. 1G) and protein (Fig. 1H) expression in PC cells were relatively higher than those in HPNE cells.

### MICAL1 promoted PC cell proliferation, invasion, and metastasis in vitro

**Fig. 2:**
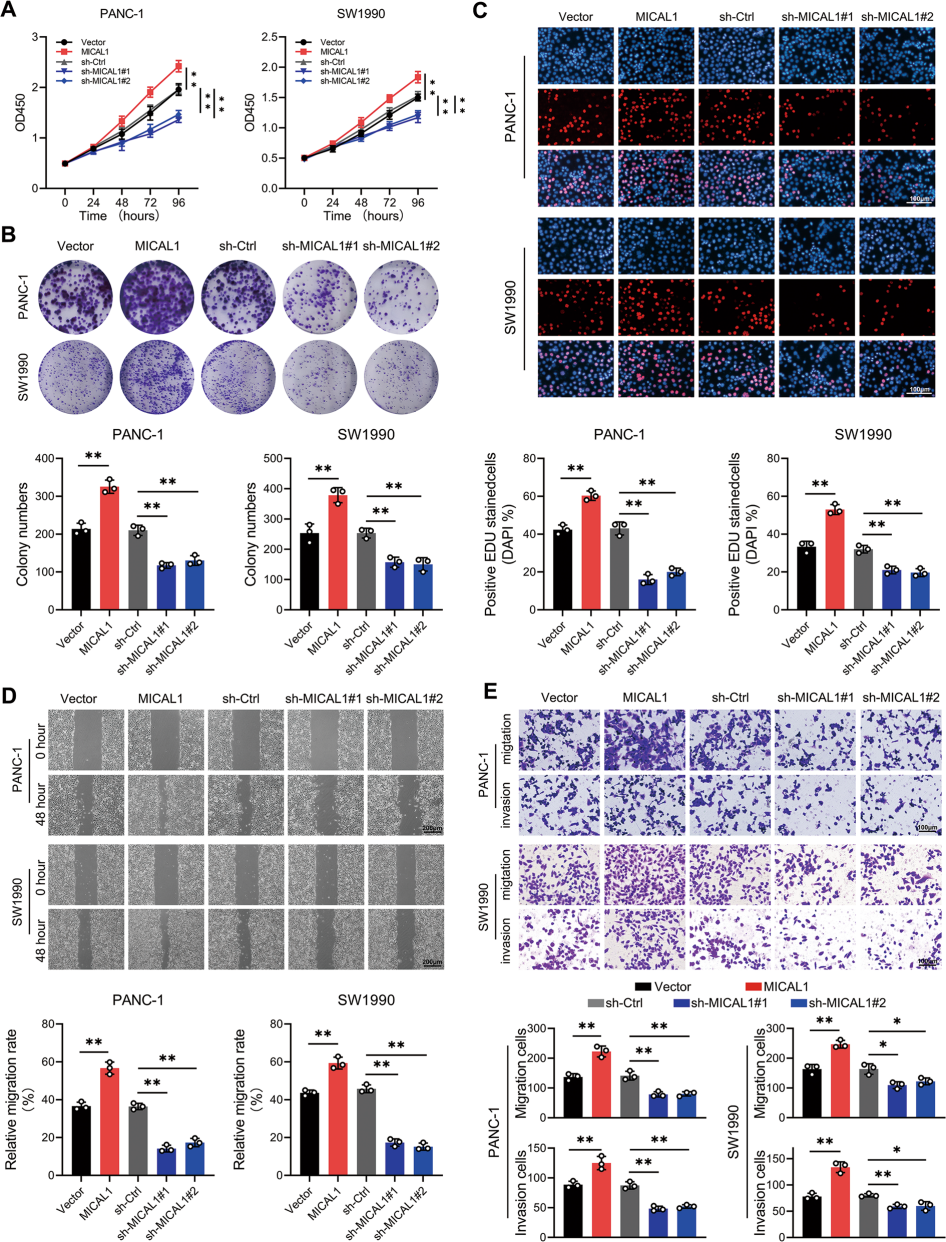
MICAL1 promoted PC cell proliferation, invasion, and metastasis in vitro. **A**–**C** Cell proliferation of PANC-1 and SW1990 cells with MICAL1 stable overexpression and MICAL1 stable silencing were determined by CCK-8 (**A**), colony formation (**B**) and EdU (**C**) assays. **D**, **E** Cell metastasis and invasion of indicated PC cells were determined by wound healing (**D**) and Transwell (**E**) assays. Data represent mean ± SD of three independent experiments and were analyzed by two-sided unpaired Student’s t-test, *P < 0.05, **P < 0.01

Stable MICAL1-overexpressing, MICAL1-silencing and their corresponding control cells of PANC-1, and SW1990 cells were constructed. As evidenced by CCK-8 (Fig. 2A), colony formation (Fig. 2B) and EdU assays (Fig. 2C), MICAL1 overexpression significantly promoted PC cell proliferation in vitro. Conversely, MICAL1 silencing suppressed cell growth. Wound healing (Fig. 2D) and Transwell (Fig. 2E) assays indicated that MICAL1 overexpression induced and MICAL1 silencing inhibited the migratory and invasive ability of PC cells.

### MICAL1 promoted PC cell proliferation and metastasis in vivo

**Fig. 3:**
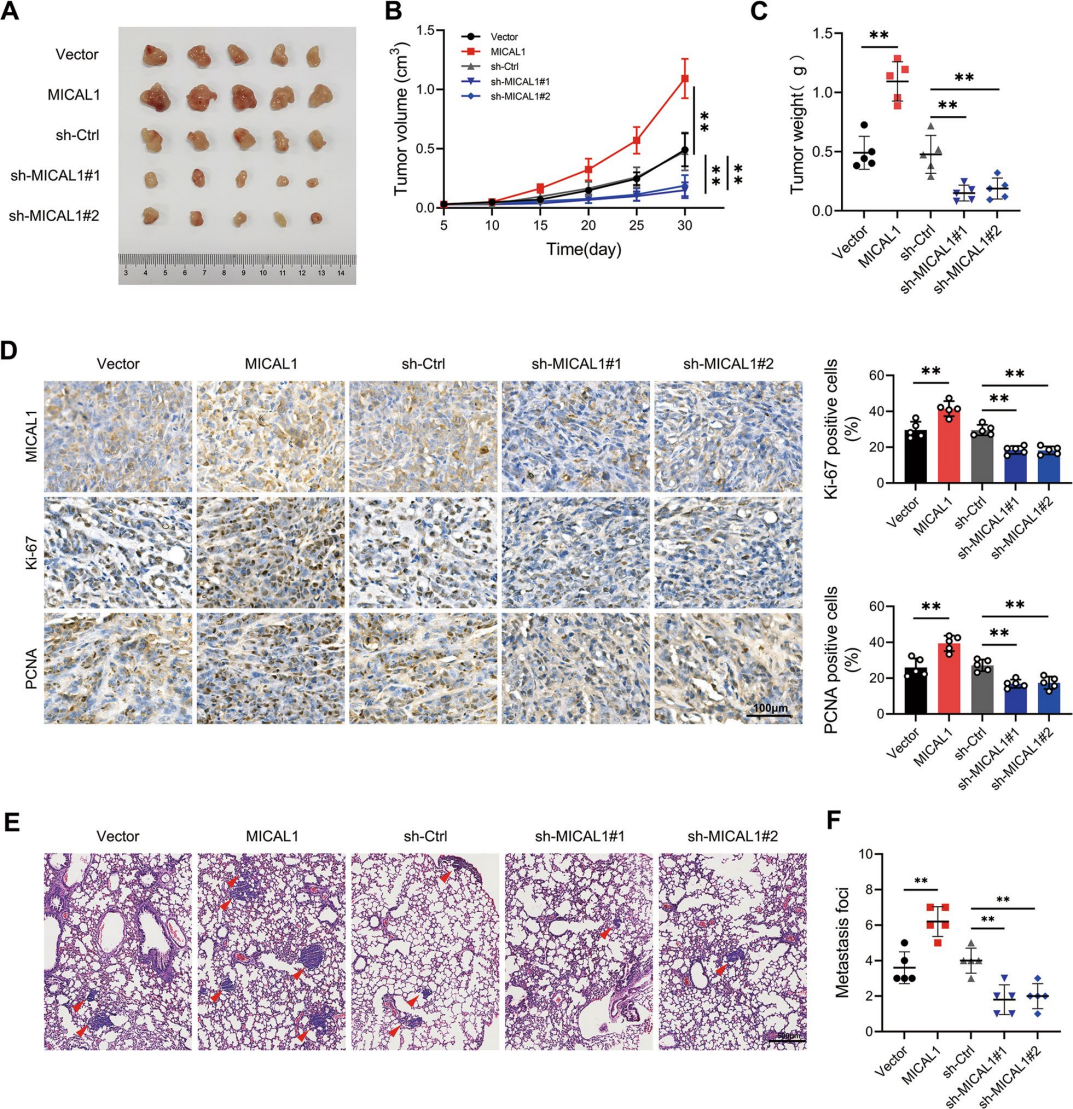
MICAL1 promoted PC cell proliferation and metastasis in vivo. **A**–**C** The tumor substances (**A**), volumes (**B**) and tumor weight (**C**) of subcutaneous tumor in different groups**. D** Representative IHC staining of MICAL1, Ki67, and PCNA in tumor tissues isolated from different nude mice groups. **E**, **F** Representative H&E (**E**) and statistic (**F**) of pulmonary metastasis focis from different nude mice groups**.** Values are means ± SD of five individuals per group (n = 5). Statistical differences were determined by two-sided Student’s t-test. *, *P* < 0.05; **, *P* < 0.01

The subcutaneous tumorigenesis model and the caudal vein pulmonary metastasis model were constructed with PANC-1 cells to investigate whether MICAL1 played a role in tumor formation and metastasis in vivo. Consistent with the in vitro results, MICAL1 overexpression promoted and MICAL1 silencing suppressed the tumorigenic ability of PC cells in vivo (Fig. 3A–C). The expression levels of two proliferation markers, Ki-67 and PCNA, showed a positive correlation with MICAL1 (Fig. 3D). The pulmonary metastasis model indicated that MICAL1 overexpression increased whereas MICAL1 silencing decreased the pulmonary metastasis foci (Fig. 3E and F).

### MICAL1 activated WNT/β-catenin signaling in PC

**Fig. 4:**
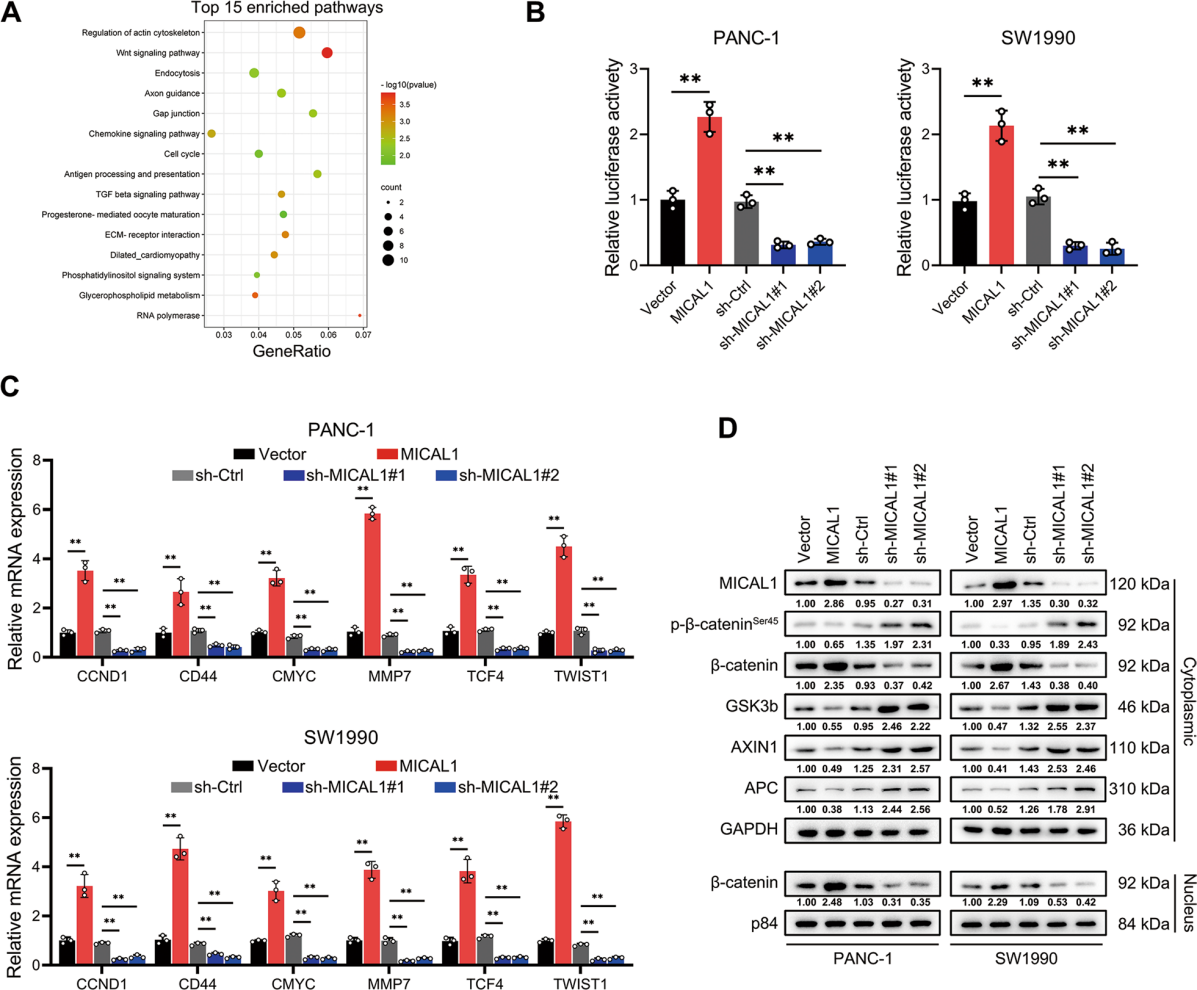
MICAL1 activated Wnt/β-catenin signaling in PC. **A** Scatter plot of top 15 enriched KEGG pathways. **B** MICAL1 stable overexpression cells, MICAL1 stable silencing cells and their respective control cells were transfected with the TOP/FOP Flash reporter plasmids and the luciferase activities were measured 48 h after transfection. **C** Established downstream target genes of the Wnt/β-catenin pathway, including CCND1, CD44, CMYC, MMP7, TCF4, and TWIST1 were detected by RT-qPCR in indicated cells. **D** Crucial proteins of WNT pathway including phosphorylation β-catenin at Ser45 site, β-catenin, GSK-3b, AXIN1, APC and intracellular β-catenin were detected by western blotting in indicated cells**.** Data represent mean ± SD of three independent experiments and were analyzed by two-sided unpaired Student’s t-test, ns = no significance, *P < 0.05, **P < 0.01

RNA-seq was used to detect differentially expressed genes (DEGs) between MICAL1-overexpressing PANC-1 cells and MICAL1-silencing PANC-1 cells at the criteria of |log_2_ FC|> 1 and *P* < 0.05. According to the result of KEGG enrichment analysis, DEGs were significantly enriched in the WNT pathway (Fig. 4A), suggesting that MICAL1 may involved in the WNT pathway. TOP/FOP flash assay indicated that MICAL1 overexpression enhanced whereas MICAL1 silencing inhibited β-catenin-dependent signaling events in PC cells (Fig. 4B). Several genes, which are related closely to tumorigenesis, progression and widely recognized downstream target of WNT/β-catenin pathway were detected by qRT-PCR. The result showed that these genes significantly increased in MICAL1-overexpressing PC cells, and reduced in MICAL1 knocked down PC cells (Fig. 4C). The protein expression layer, MICAL1 overexpression significantly suppressed the expression of GSK3b, AXIN1, APC and Ser45-phosphorylated β-catenin while increasing the expression of β-catenin in both cytoplasm and nucleus (Fig. 4D). MICAL1 knocked down showed the opposite result.

### MICAL1 promoted the progression of PC by activating WNT pathway

To further verify whether the activation of the WNT pathway plays a partial role in MICAL1 promoted progression of PC, WNT inhibitors, KYA1797K (KYA) and IWR-1, were applied. Inhibitor group cells were precultured in complete medium containing either KYA (0.75 µM) or IWR-1 (10 µM) for 48 h, and maintaining this concentration until the experiments ended. Assays, including CCK8, colony formation, EdU, Transwell and wound healing (Additional file 1: Fig. S1A–E), consistently showed that WNT inhibitors could reverse the phenotypes induced by MICAL1 overexpression. The elevated WNT/β-catenin competence also presented a downtrend when treated with WNT inhibitors (Additional file 1: Fig. S1F). These data suggest that MICAL1 promotes the progression of PC partly by activating WNT pathway.

### MICAL1 promoted WNT pathway by promoting phosphorylation of TBC1D1

**Fig. 5:**
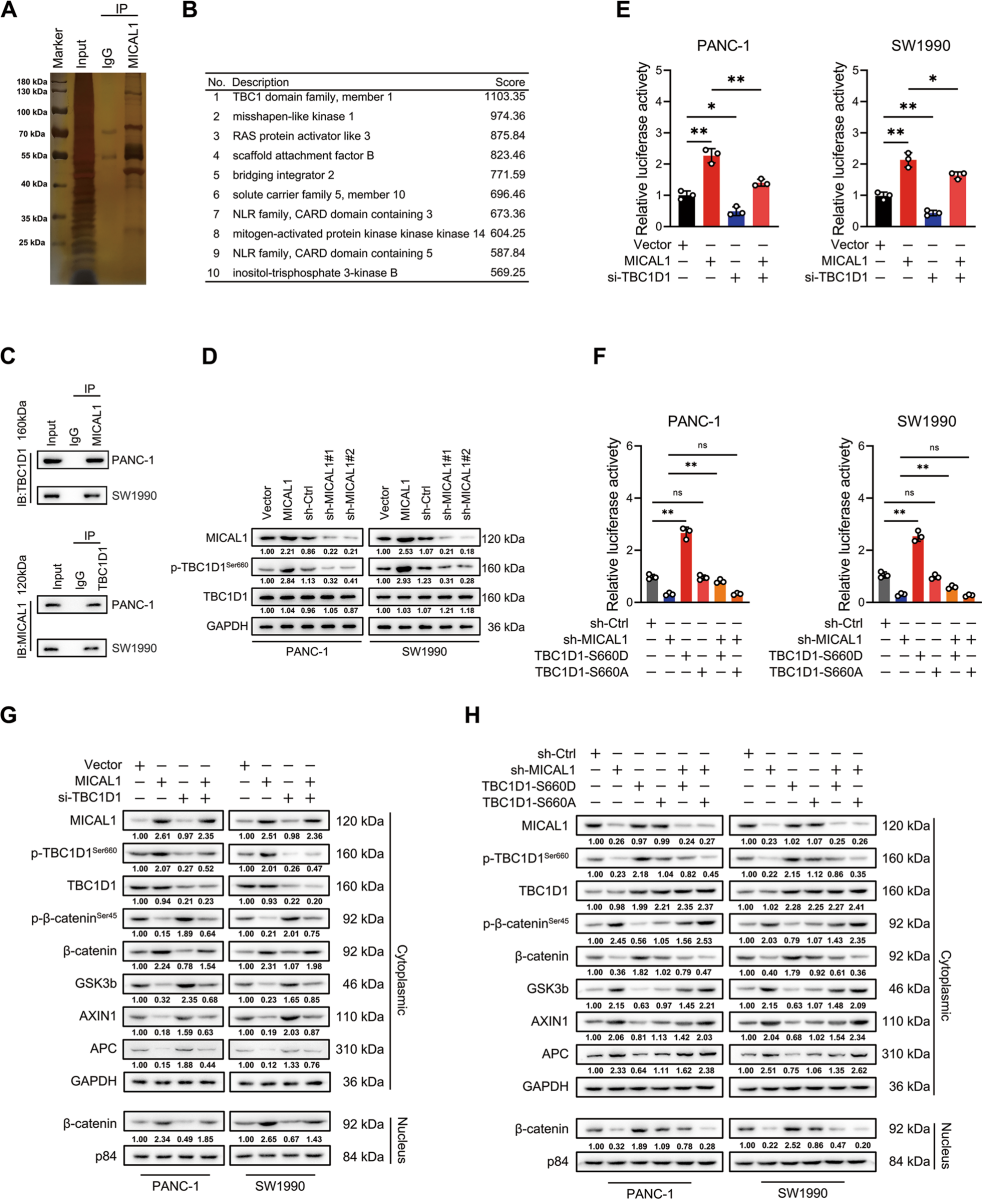
MICAL1 promoted WNT pathway by phosphorylating TBC1D1. **A**, **B** Silver staining (**A**) and proteomics mass spectrometry analysis (**B**) of the proteins associated with MICAL1**. C** Endogenous immunoprecipitation assays were conducted to verify the inosculation between MICAL1 and TBC1D1. **D** TBC1D1 and phosphorylated TBC1D1 at Ser660 site were detected in indicated PC cells. **E**, **F** TOP/FOP flash assays were conducted to evaluate the effect of TBC1D1 silencing (**E**) or Ser660 site phosphorylation of TBC1D1 (**F**) on β-catenin-dependent signaling events**. H**, **I** Western blot analysis was conducted to evaluate the effect of TBC1D1 silencing (**H**) or corresponding plasmid transfection (**I**) on key proteins of WNT pathway

To investigate how MICAL1 activates WNT, proteins co-immunoprecipitated with MICAL1 were separated by 10% protein gel and stained with silver (Fig. 5A). Proteomics mass spectrometry results showed that the TBC domain family member 1 (TBC1D1) got the highest binding score (Fig. 5B). Endogenous and exogenous immunoprecipitation assays confirmed that MICAL1 associated with TBC1D1 (Fig. 5C and Additional file 2: Fig. S2A). Whereas MICAL1 overexpression did not change the total amount of TBC1D1 protein, it increased the expression abundance of phosphorylation of TBC1D1 at Ser660 site (Fig. 5D) and this effect could not be inhibited by using F-actin disrupter agent cyclochalasin D (Additional file 2: Fig. S2B). Therefore, we speculated that MICAL1 may activate WNT pathway by TBC1D1, more specifically, by promoting phosphorylation of TBC1D1 at Ser660 site. Therefore, TOP/FOP flash were performed again. The results showed that MICAL1 overexpression enhanced WNT pathway activity but declined when TBC1D1 gene was knocked down (Fig. 5E). MICAL1 knocked down reduced WNT pathway activity elevated when pseudo-phosphorylated mutant of TBC1D1 at Ser660 instead of Ser660 nonphosphorylable mutant type plasmids of TBC1D1 was transfected (Fig. 5F). The detection of crucial proteins (Fig. 5G and H) and target genes (Additional file 2: Fig. S2B and C) of Wnt/β-catenin pathway led to the same conclusion. These results indicated that MICAL1 promoted WNT pathway by promoting the phosphorylation of TBC1D1 at Ser660.

### Ser660 phosphorylated TBC1D1 promoted FZD7 membranal localization and inhibited FZD7 lysosomal degradation

**Fig. 6:**
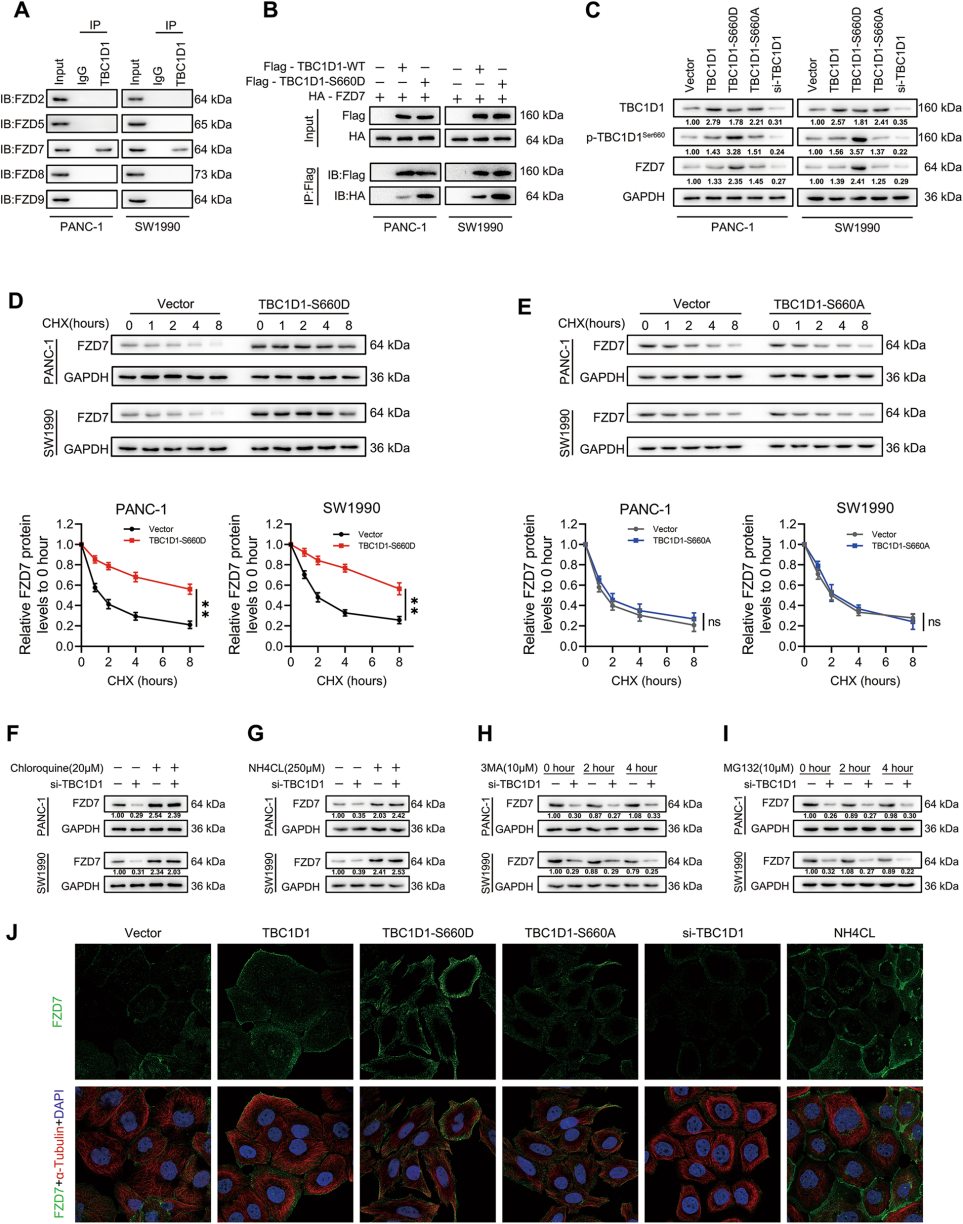
p-TBC1D1^Ser660^ promoted FZD7 membranal localization and inhibited FZD7 lysosomal degradation. **A** Screening the incorporable FZDs with TBC1D1 by immunoprecipitation assay. **B** The inosculation between TBC1D1 and FZD7 was detected with or without pseudo-phosphorylated mutant type plasmids of TBC1D1 at Ser660 site transfection. **C** Western blot analysis was conducted to evaluate the effect of TBC1D1 Ser660 phosphorylation or silencing on the expression of FZD7. **D**, **E** Cycloheximide (CHX)-chase assay showing the effects of pseudo-phosphorylated (**D**) or nonphosphorylatable (**E**) plasmids of TBC1D1 at Ser660 site on the decline of FZD7 in PC cells. Data represent mean ± SD of three independent experiments and were analyzed by two-sided unpaired Student’s t-test, *P < 0.05, **P < 0.01. **F**–**I** Western blot analysis was conducted to evaluate the effect of TBC1D1 on FZD7 expression in the absence and presence of lysosomal inhibitors chloroquine (**F**) and NH4Cl (**G**), autophagy inhibitor 3-MA (**H**) and proteasome inhibitor MG132 (**I**). **J** The detection of FZD7 expression in corresponding groups by immunofluorescence

New studies suggest that the localization and stability of seven transmembrane receptors-Frizzled (FZDs) play a crucial role in determining Wnt function [22–24]. Immunoprecipitation assays indicated that TBC1D1 could associate with FZD7 (Fig. 6A) and this bonding ability was enhanced when pseudo-phosphorylated mutant type plasmids of TBC1D1 at Ser660 site were transfected (Fig. 6B). In addition, western blot analysis indicated that when pseudo-phosphorylated plasmids were transfected, the protein abundance of FZD7 increased (Fig. 6C). However, transfection of pseudo-phosphorylated mutant type plasmids did not affect the mRNA expression of FZD7 (Additional file 3: Fig. S3A),it alleviated the degradation of FZD7 protein when the cells were treated with cycloheximide (CHX, 50 μM) (Fig. 6D). However, there was no significant difference in the effect of transfection of nonphosphorylatable plasmids (TBC1D1-S660A) on the degradation rate of FZD7 (Fig. 6E). In addition, both selective lysosomal inhibitors NH_4_Cl (250 μM) and chloroquine (20 μM) attenuated the downregulation of FZD7 by TBC1D1-silencing (Fig. 6F, G). However, the effect of TBC1D1-silencing on FZD7 was not affected by the inhibitors for autophagy 3-MA and proteasome MG132 (Fig. 6H, I). The results of immunofluorescence experiment also showed that transfection of TBC1D1-S660D and lysosomal inhibitor treatment could promote the expression of FZD7 in cell membrane, while transfection of TBC1D1-S660A had no significant difference with the control group (Fig. 6J). These findings suggested that phosphorylation of TBC1D1 at Ser660 site suppresses the degradation of FZD7 in lysosomes.

### p-TBC1D1^Ser660^ promoted malignant activities could be inhibited by FZD7 interference

**Fig. 7:**
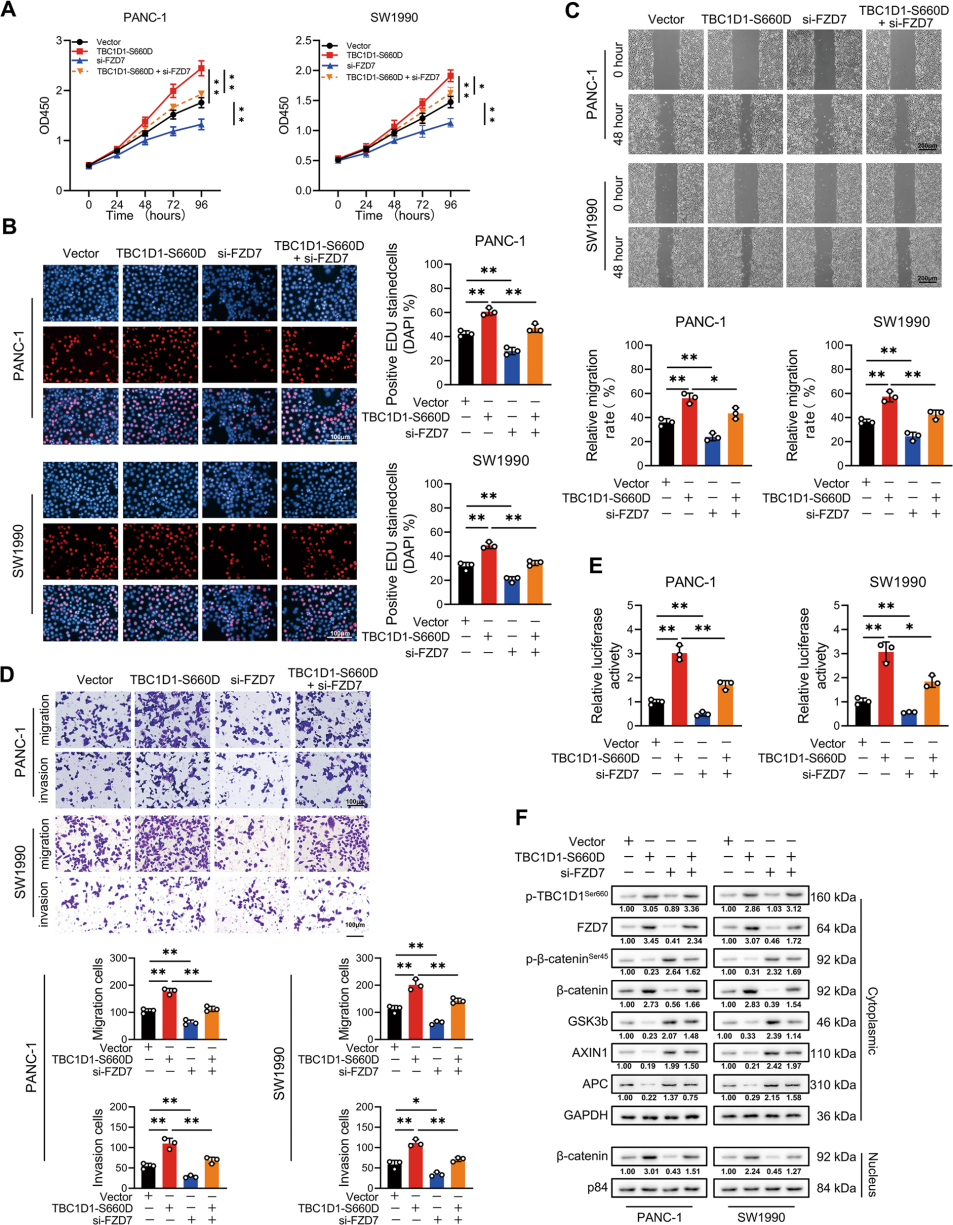
p-TBC1D1^Ser660^ promoted malignant activities could be inhibited by FZD7 interference. **A**, **B** Cell proliferative capability was determined by CCK-8 (**A**) and EdU (**B**) assays. **C**, **D** Cell metastatic and invasive capability were determined by wound healing (**C**) and Transwell (**D**) assays**. E** TOP/FOP flash assay was conducted on indicate cells to evaluate the effect of FZD7 on WNT/β-catenin signaling. **F** Western blot analysis was conducted on indicated cells to evaluate the effect of FZD7 on WNT/β-catenin signaling. Data represent mean ± SD of three independent experiments and were analyzed by two-sided unpaired Student’s t-test, *P < 0.05, **P < 0.01

As shown in Fig. 7A–C, the increased cell proliferation in PC cells by transfection of pseudo-phosphorylated mutant type plasmids of TBC1D1 at Ser660 site were distinctly reduced by concomitant suppression of FZD7. FZD7 inhibition consistently downgraded the enhanced ability of invasion and metastasis induced by the transfection of pseudo-phosphorylated plasmids in PC cells (Fig. 7D, E). Transfection of pseudo-phosphorylated plasmids promoted catenin entrance into the nucleus but was restrained when FZD7 was inhibited. Meanwhile, the retardation of TBC1D1-silencing could also be expedited by overexpression of FZD7 (Additional file 4: Fig. S4). Therefore, p-TBC1D1^Ser660^ promoted the PC progression by FZD7.

### MICAL1 expression was positively correlated with p-TBC1D1^Ser660^, FZD7 and β-catenin

**Fig. 8:**
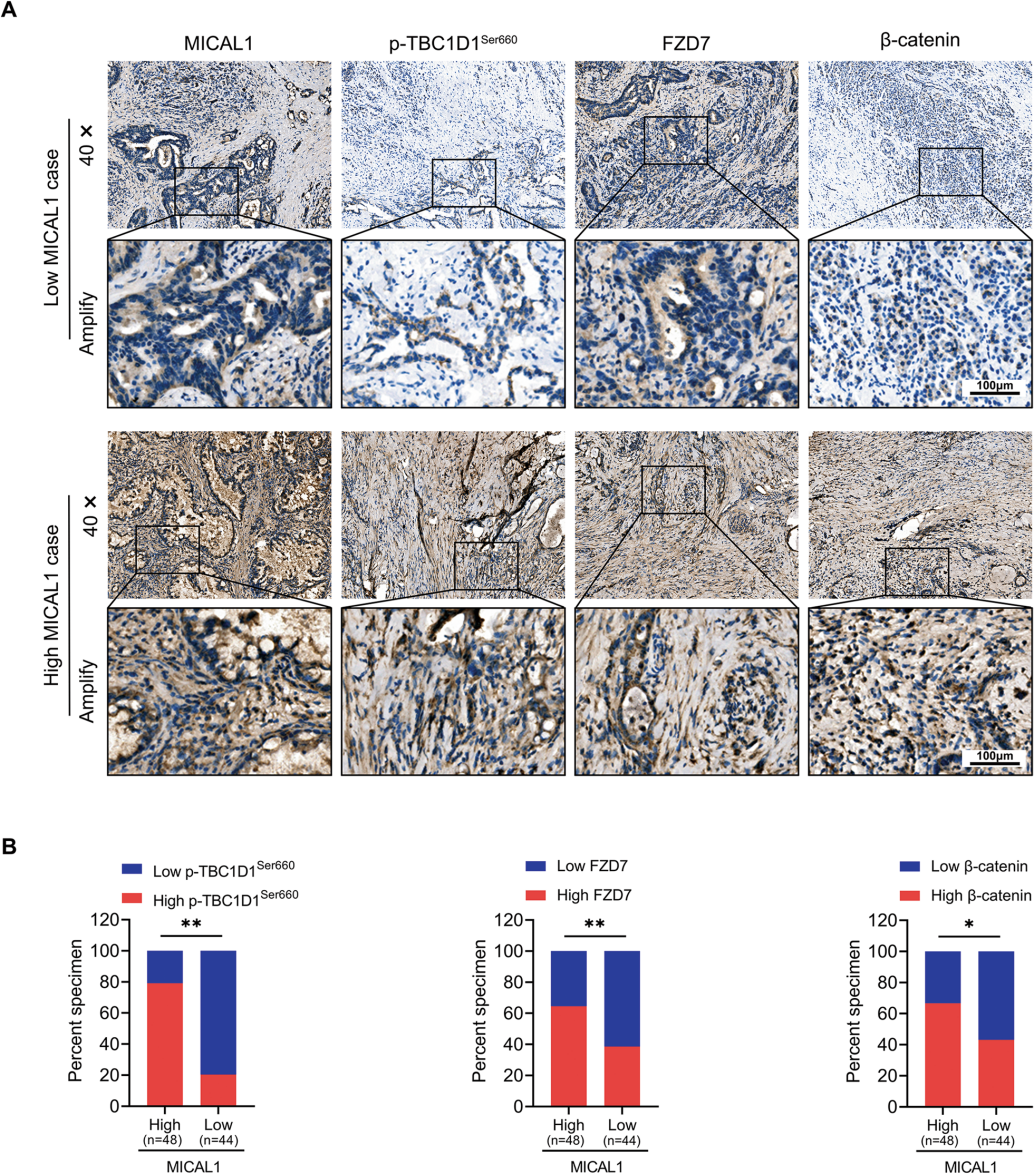
MICAL1 expression was positively correlated with p-TBC1D1 ^Ser660^, FZD7 and β-catenin. **A** Representative immunohistochemical staining of MICAL1, p-TBC1D1^Ser660^, FZD7 and β-catenin in sections of pancreatic cancer specimens. **B** Immunohistochemical analysis of MICAL1, p-TBC1D1.^Ser660^, FZD7 and β-catenin expression in specimens from 92 cases of pancreatic cancer. Statistical differences were determined by Chi-squared Test. *, *P* < 0.05; **, *P* < 0.01

The IHC assays showed relatively decreased intensity of p-TBC1D1^Ser660^, FZD7 and β-catenin expression when expression of MICAL1 was relatively low in human PC specimens. In contrast, increased intensity of p-TBC1D1 ^Ser660^, FZD7 and β-catenin expression were detected when the expression of MICAL1 in human PC specimens was high (Fig. 8).

## Discussion

**Fig. 9:**
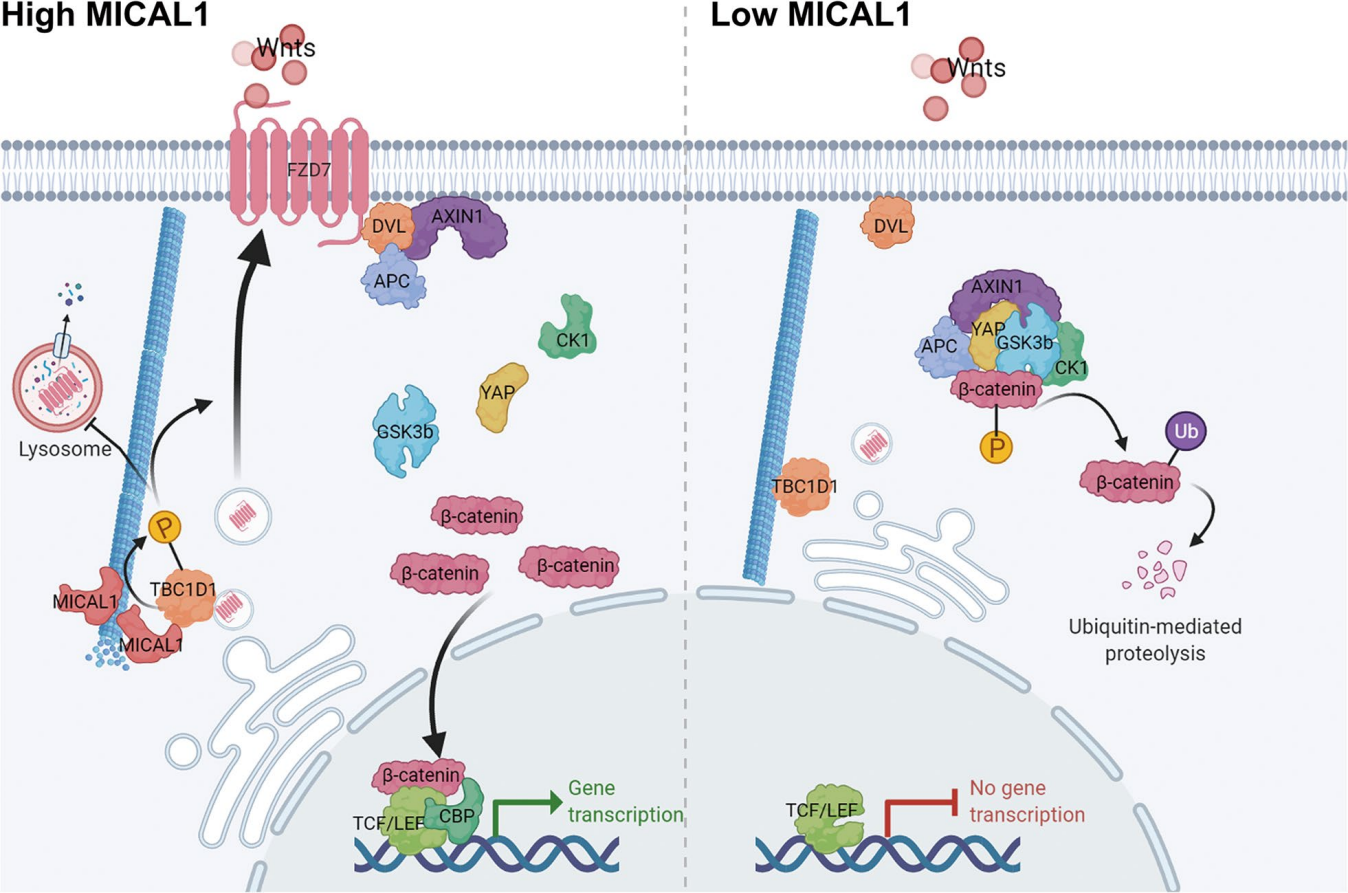
Proposed mechanistic scheme of MICAL1 in promoting WNT/β-catenin pathway in pancreatic cancer, created with BioRender.com (Agreement number: HP24M38W97)

Despite decades of research focusing on it, PC is still a leading cause of cancer-related mortality worldwide [25]. Therefore, the identification of novel prognostic biomarkers and treatment targets of PC is urgent. In this study, we found that MICAL1 increased in PC tissues compared with adjacent normal tissues and this amplification was related to poor clinical prognosis of PC. Furthermore, our results indicated that MICAL1 (1) affected the intracellular distribution of FZD7 by promoting TBC1D1 phosphorylation, (2) activated the WNT pathway, and (3) ultimately exerted an auxo-action of PC (Fig. 9).

MICAL1 could weaken the links between actin subunits and enhance the interaction with actin-severing proteins such as cofilin1, thereby playing a central and prominent role in cytoskeleton regulation [26]. Accompanied by this dynamic actin cytoskeleton remodeling, MICAL1 participates in various key cellular functions, such as membrane trafficking [27], EMT [11], cell viability [10], immunity [28], and tumorigenesis [29]. MICAL1 plays diverse roles in different tumors. MICAL1 could inhibit colorectal cancer by regulating EGR1 [11]. However, MICAL1 could also promote tumor progression in gastric cancer [17], melanoma [30], and breast cancer [9, 10, 12]. In this study, enhanced expression of MICAL1 in PC was discovered, which was associated with poor prognosis. Moreover, in vitro and in vivo studies showed that MICAL1 promoted the proliferation, invasion and metastasis of PC. RNA-seq and KEGG pathway enrichment analysis indicated that the genes altered by MICAL1 overexpression in PANC-1 were enriched in the WNT pathway, suggesting that the WNT pathway is vital for MICAL1 to promote PC progression. This hypothesis was supported by further experiments such as evaluation of β-catenin-dependent signaling events by TOP/FOT flash assay, detection of Wnt/β-catenin downstream target by qRT-PCR, and analysis of β-catenin distribution by the western blot.

Further experimental results showed that MICAL1 interacted with TBC1D1 and promoted its phosphorylation at Ser660 site. TBC1 domain family members are characterized by the presence of highly conserved Tre2–Bub2–Cdc16 (TBC) domain and function as Rab GTPase-activating proteins (GAPs) [31]. GAPs contain multiple phosphorylation sites for Akt and AMPK. When GAPs were phosphorylated by those Ser/Thr kinases, the inhibitory action on their target Rab GTPases ceased [32]. Rab GTPases are key determinants in the generation and/or destination of all events in intracellular traffic [33]. Numerous previous studies have shown that phosphorylation of TBC1D1 is involved in the release of GLUT4 storage vesicles and their loading onto microtubule motors for transport to the cell surface [34–36], and knock-down of TBC1D1 results in greatly reduced insulin-stimulated glucose uptake due to compromised trafficking of the GLUT4 transporter.However, knock-down of TBC1D1 also results in decreased palmitate uptake and oxidation in both isolated skeletal muscle and cultured muscle cells [37]. Different RabGTPasses participate in TBC1D1 mediated intelligent traffic may account for this division [38]. In this study, immunofluorescence and Western Blot experiments showed that transfection of pseudo-phosphorylated mutant type plasmids of TBC1D1 at Ser660 site inhibited the degradation of FZD7 in lysosomes and promoted its cell membrane distribution. At the same time, knock-down of TBC1D1 expression achieved the opposite result. Based on the above experimental results, we inferred from this that TBC1D1 involved in the release of FZD7 storage vesicles and their loading onto microtubule motors for transport to the cell surface.

FZDs belong to the superfamily of G protein-coupled receptor (GPCR) and function as main receptors of the WNT pathway [39]. The binding between Wnts and FZDs is an initial step in WNT pathway activation, so the localization and stability of FZDs play a crucial role in determining WNT function [40]. However, the mechanism through which FZDs accumulate on the cytomembrane remains unclear. The immunoprecipitation assay indicated that FZD7, which is universally upregulated in cancer, could associate with TBC1D1. The phosphorylation of TBC1D1 on the Ser660 site enhanced this combination, inhibited the lysosomal degradation and promoted the distribution of FZD7 in the cytomembrane. This re-distribution of FZD7 seems to have (1) promoted the binding with Wnts, promoted the activation of the WNT pathway, (2) facilitated the entrance of β-catenin into the nucleus, (3) initiated the transcription of downstream target genes, and (4) ultimately promoted the proliferation, invasion, and metastasis of PC.

## Conclusions

Overall, our results indicated that MICAL1 played a tumor-promoting role in PC. It facilitated the proliferation invasion and metastasis capacity of PC cells in vivo and in vitro. Targeting MICAL1 may be a potential therapeutic strategy for PC.

## Supplementary Information

**Additional file 1: Figure S1.** MICAL1 promoted the progression of PC by activating WNT pathway. **A**–**C** Cell proliferation of indicated PC cells processed or untreated with WNT inhibitors were determined by CCK-8(**A**), colony formation (**B**) and EdU (**C**) assays. **D**, **E** Cell metastasis and invasion of indicated PC cells processed or untreated with WNT inhibitors were determined by Transwell (**D**) and wound healing (**E**) assays. **F** Crucial proteins of WNT pathway were detected by western blot in indicated cells processed or untreated with WNT inhibitors. Data represent mean ± SD of 3 independent experiments and were analyzed by two-sided unpaired Student t test, *P < 0.05, **P < 0.01.

**Additional file 2: Figure S2.** MICAL1 promoted the transcription of target genes by phosphorylating TBC1D1. **A** Exogenous immunoprecipitation assays confirmed that MICAL1 associated with TBC1D1. **B** Western blot was used to detect the expression of TBC1D1 phosphorylated at Ser660 site in cells treated with cytochalasin D (10uM). **C** Target genes of WNT/β-catenin pathway were detected in MICAL1 overexpression PC cells with or without interference of TBC1D1. **D** Target genes of WNT/β-catenin pathway were detected in MICAL1 repression PC cells co-transfection with different phosphorylated TBC1D1.

**Additional file 3: Figure S3.** phosphorylating TBC1D1 did not affect the transcription of FZD7 **A** The mRNA level of FZD7 was detected in PC cells when TBC1D1 expression level or phosphorylation level changed.

**Additional file 4: Figure S4.** TBC1D1 interference inhibited malignant activities could be rescued by FZD7 reestablishment. **A**–**B** Cell proliferative capability was determined by CCK-8(**A**) and EdU (**B**)assays. **C**–**D** Cell metastatic and invasive capability were determined by wound healing (**C**) and Transwell (**D**) assays. **E **TOP/FOP flash assay was conducted on indicate cells to evaluate the effect of FZD7 on WNT/β-catenin signaling. **F** Western blot assay was conducted on indicate cells to evaluate the effect of FZD7 on WNT/β-catenin signaling. Data represent mean ± SD of 3 independent experiments and were analyzed by two-sided unpaired Student t test, *P < 0.05, **P < 0.01.

**Additional file 5: Table S1. **Target sequences of relevant genes used.** Table S2.** Primer sequences of relevant genes used. **Table S3.** Antibodies and dilution ratios of relevant genes used.

## Data Availability

The datasets used and/or analyzed during the current study are available from the corresponding author on reasonable request. All data generated or analyzed during this study are included in this published article [and its Additional information files].

## Abbreviations

PC: Pancreatic cancer
MICAL: Molecules interacting with casL
Wnts: WNT glycoproteins
FZD: Frizzled
GEPIA: Gene Expression Profiling Interactive Analysis
GEO: Gene Expression Omnibus
siRNAs: Small interfering RNAs
BSA: Bovine serum albumin
IHC: Immunohistochemistry
PFA: Paraformaldehyde
DEGs: Differentially expressed genes
CHX: Cycloheximide
GAPs: GTPase-activating proteins
GPCR: G protein-coupled receptor
