# Supplementary figures and images for: MICAL1 facilitates pancreatic cancer proliferation, migration, and invasion by activating WNT/β-catenin pathway
Source: J Transl Med. 2022 Nov 12;20:528. doi: 10.1186/s12967-022-03749-1 (PMC9652939; doi:10.1186/s12967-022-03749-1)

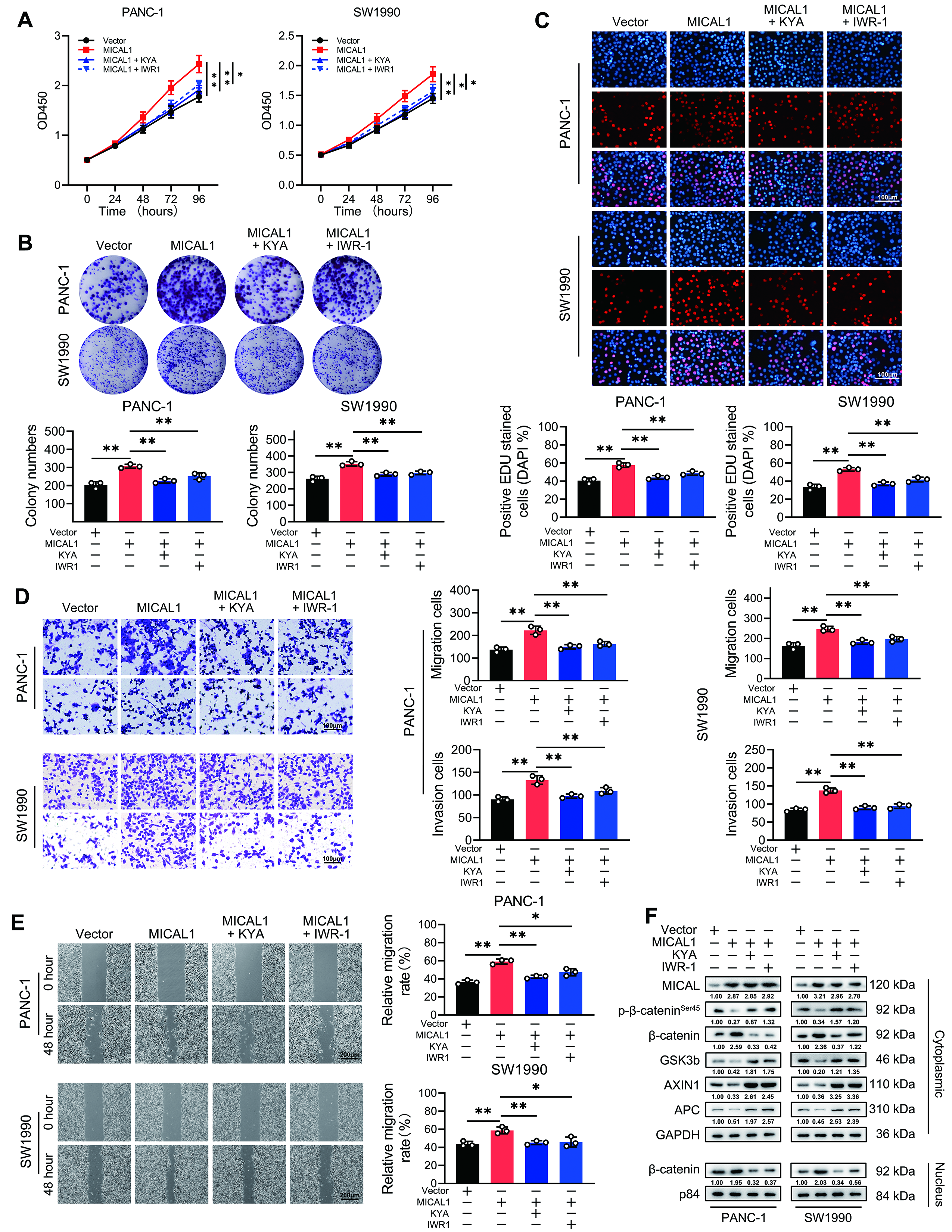

Supplement: Supplementary file 1 — Additional file 1: Figure S1. MICAL1 promoted the progression of PC by activating WNT pathway. A–C Cell proliferation of indicated PC cells processed or untreated with WNT inhibitors were determined by CCK-8(A), colony formation (B) and EdU (C) assays. D, E Cell metastasis and invasion of indicated PC cells processed or untreated with WNT inhibitors were determined by Transwell (D) and wound healing (E) assays. F Crucial proteins of WNT pathway were detected by western blot in indicated cells processed or untreated with WNT inhibitors. Data represent mean ± SD of 3 independent experiments and were analyzed by two-sided unpaired Student t test, *P < 0.05, **P < 0.01. [file 12967_2022_3749_MOESM1_ESM.tif]

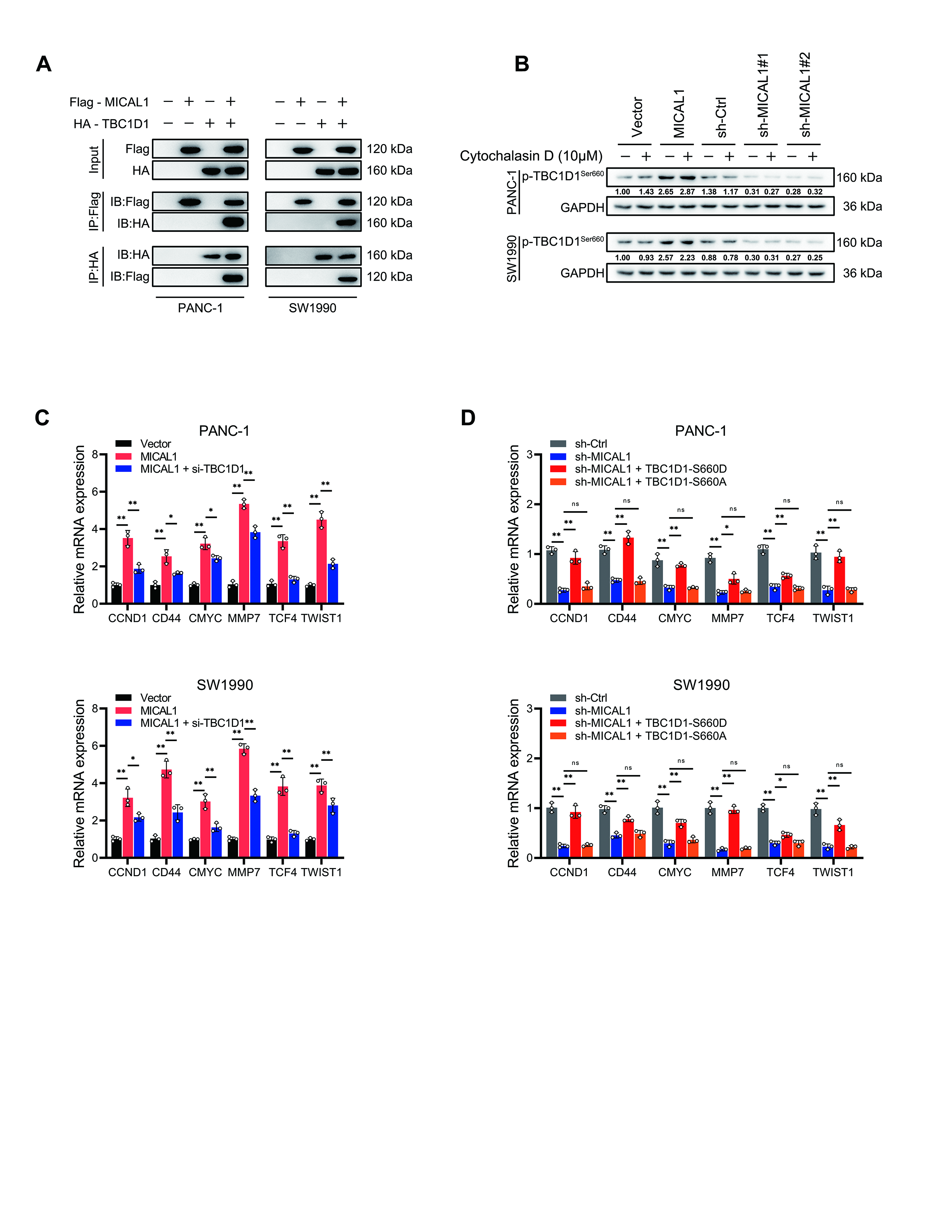

Supplement: Supplementary file 2 — Additional file 2: Figure S2. MICAL1 promoted the transcription of target genes by phosphorylating TBC1D1. A Exogenous immunoprecipitation assays confirmed that MICAL1 associated with TBC1D1. B Western blot was used to detect the expression of TBC1D1 phosphorylated at Ser660 site in cells treated with cytochalasin D (10uM). C Target genes of WNT/β-catenin pathway were detected in MICAL1 overexpression PC cells with or without interference of TBC1D1. D Target genes of WNT/β-catenin pathway were detected in MICAL1 repression PC cells co-transfection with different phosphorylated TBC1D1. [file 12967_2022_3749_MOESM2_ESM.tif]

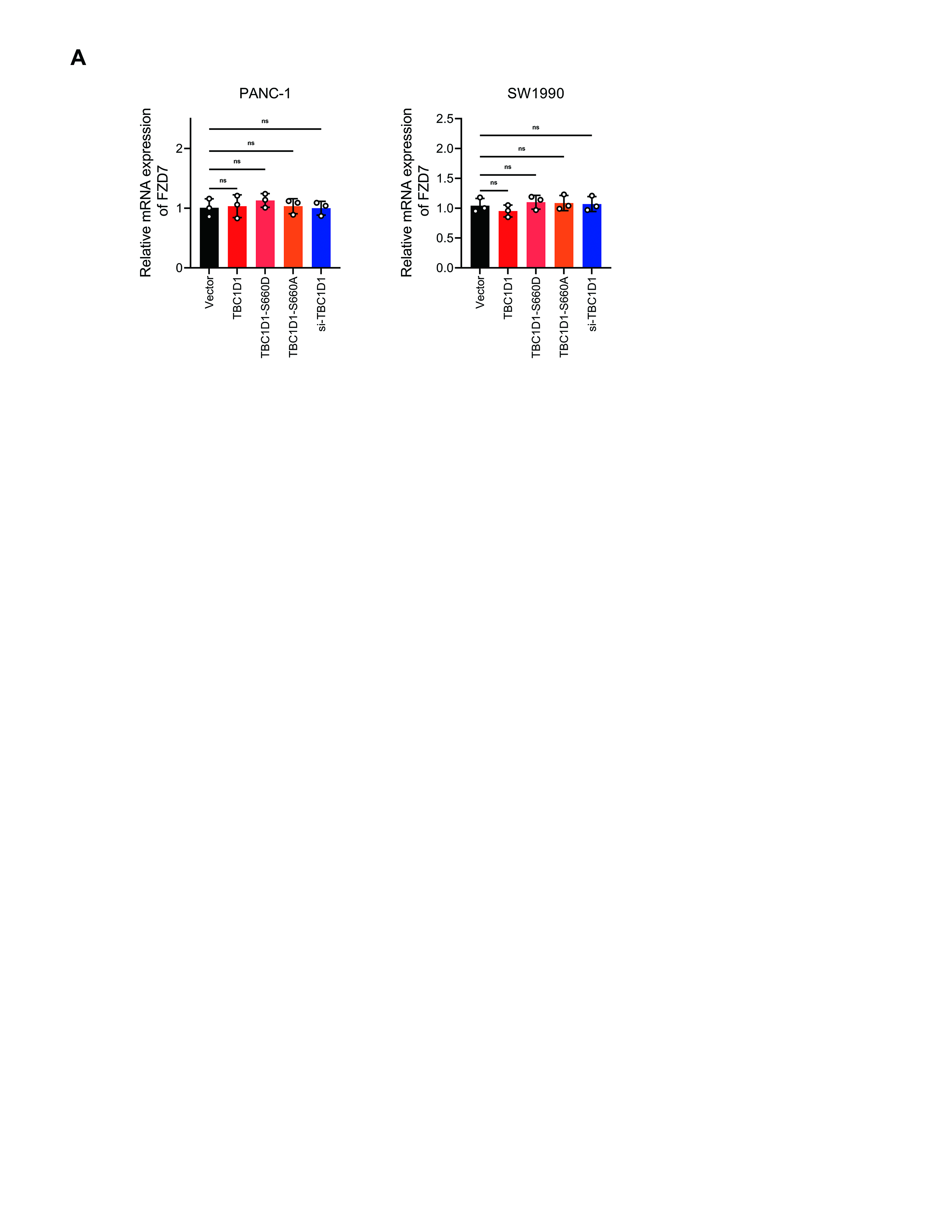

Supplement: Supplementary file 3 — Additional file 3: Figure S3. phosphorylating TBC1D1 did not affect the transcription of FZD7 A The mRNA level of FZD7 was detected in PC cells when TBC1D1 expression level or phosphorylation level changed. [file 12967_2022_3749_MOESM3_ESM.tif]

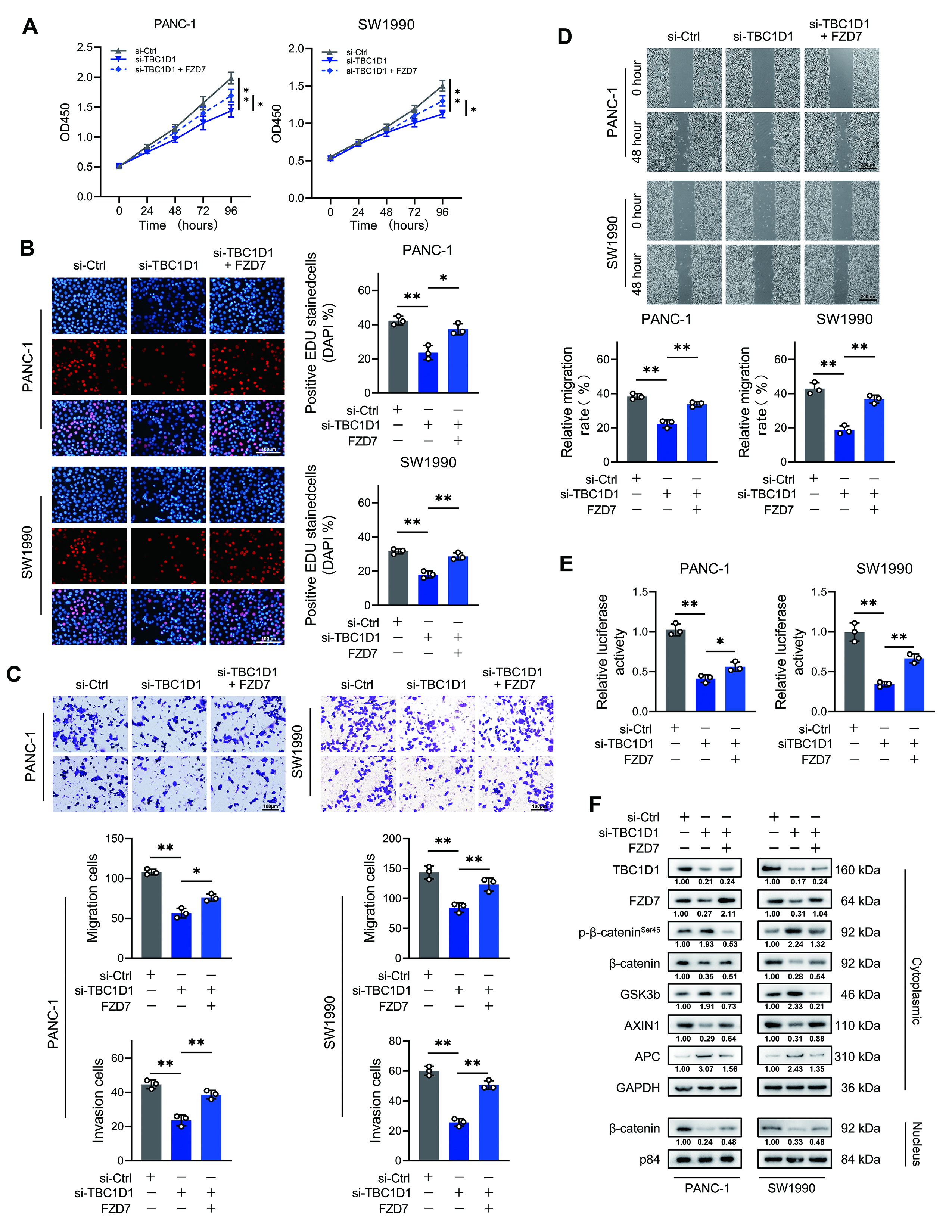

Supplement: Supplementary file 4 — Additional file 4: Figure S4. TBC1D1 interference inhibited malignant activities could be rescued by FZD7 reestablishment. A–B Cell proliferative capability was determined by CCK-8(A) and EdU (B)assays. C–D Cell metastatic and invasive capability were determined by wound healing (C) and Transwell (D) assays. E TOP/FOP flash assay was conducted on indicate cells to evaluate the effect of FZD7 on WNT/β-catenin signaling. F Western blot assay was conducted on indicate cells to evaluate the effect of FZD7 on WNT/β-catenin signaling. Data represent mean ± SD of 3 independent experiments and were analyzed by two-sided unpaired Student t test, *P < 0.05, **P < 0.01. [file 12967_2022_3749_MOESM4_ESM.tif]
