# Supplementary material for: MICAL1 facilitates pancreatic cancer proliferation, migration, and invasion by activating WNT/β-catenin pathway
Source: J Transl Med. 2022 Nov 12;20:528. doi: 10.1186/s12967-022-03749-1 (PMC9652939; doi:10.1186/s12967-022-03749-1)
Supplement: Supplementary file 5 — Additional file 5: Table S1. Target sequences of relevant genes used. Table S2. Primer sequences of relevant genes used. Table S3. Antibodies and dilution ratios of relevant genes used. [file 12967_2022_3749_MOESM5_ESM.docx]

**Additional file Tab.S1** Target sequences of relevant genes used.

| **Gene** | **Targeting sequences** |
| --- | --- |
| sh-MICAL1#1 | 5′-GCTGGCCAACTATGAATTTGA-3′ |
| sh-MICAL1#2 | 5′-GCATTGATCTGGAGAACATTG-3′ |
| si-FZD7 | 5′-AGTACCTGATGACCATGAT-3′ |
| si-TBC1D1 | 5′-CCAAGTTACTTTGCTTGTCTGATTA-3′ |

**Additional file Tab.S2** Primer sequences of relevant genes used.

| **Gene** | **Sequence（5′ ->3′）** | |
| --- | --- | --- |
| MICAL1 | Forward Primer | GGCACTCGGTGCTAAGAAGTT |
|  | Reverse Primer | CCCCAGTGAATTTCCACCCC |
| GAPDH | Forward Primer | CTCCAAAATCAAGTGGGGCG |
|  | Reverse Primer | TGGTTCACACCCATGACGAA |
| FZD7 | Forward Primer | GTGCCAACGGCCTGATGTA |
|  | Reverse Primer | AGGTGAGAACGGTAAAGAGCG |
| CCND1 | Forward Primer | GCTGCGAAGTGGAAACCATC |
|  | Reverse Primer | CCTCCTTCTGCACACATTTGAA |
| CD44 | Forward Primer | CTGCCGCTTTGCAGGTGTA |
|  | Reverse Primer | CATTGTGGGCAAGGTGCTATT |
| CMYC | Forward Primer | GGCTCCTGGCAAAA GGTCA |
|  | Reverse Primer | CTGCGTAGTTGTGCTG ATGT |
| MMP7 | Forward Primer | GAGTGAGCTACAGTGGGAACA |
|  | Reverse Primer | CTATGACGCGGGAGTTTAACAT |
| TCF4 | Forward Primer | CAAGCACTGCCGAC TACAATA |
|  | Reverse Primer | CCAGGCTGATTCATCCC ACTG |
| TWIST1 | Forward Primer | GTCCGCAGTCTTACGAGGAG |
|  | Reverse Primer | GCTTGAGGGTCTGAATCTTGCT |

**Additional file Tab.S3** Antibodies and dilution ratios of relevant genes used.

| **Gene** | **Brand** | **Catalog number** | **Dilution ratio** | | | |
| --- | --- | --- | --- | --- | --- | --- |
|  |  |  | **WB** | **IHC** | **IP** | **IF** |
| MICAL1 | Proteintech | 14818-1-AP | 1:1000 | 1:100 | 1:200 |  |
| GAPDH | Proteintech | 60004-1-Ig | 1:20000 |  |  |  |
| Ki-67 | Proteintech | 27309-1-AP |  | 1:1000 |  |  |
| PCNA | Proteintech | 10205-2-AP |  | 1:200 |  |  |
| p-β-catenin^Ser45^ | Cell Signaling | 9564S | 1:1000 |  |  |  |
| β-catenin | Cell Signaling | 8480 | 1:1000 | 1:50 |  |  |
| GSK3b | Cell Signaling | 12456 | 1:1000 |  |  |  |
| AXIN1 | Cell Signaling | 2074 | 1:1000 |  |  |  |
| APC | Cell Signaling | 2504 | 1:1000 |  |  |  |
| p-84 | Abcam | ab487 | 1:1000 |  |  |  |
| TBC1D1 | Proteintech | 22124-1-AP | 1:1000 |  | 1:50 |  |
| p-TBC1D1^Ser660^ | Proteintech | 29034-1-AP | 1:500 | 1:50 |  |  |
| Flag | Cell Signaling | 14793 | 1:1000 |  | 1:50 |  |
| HA | Cell Signaling | 3724 | 1:1000 |  | 1:50 |  |
| FZD2 | Proteintech | 24272-1-AP | 1:1000 |  |  |  |
| FZD5 | Proteintech | 21519-1-AP | 1:1000 |  |  |  |
| FZD7 | Proteintech | 16974-1-AP | 1:1000 | 1:200 |  | 1:50 |
| FZD8 | Proteintech | 55093-1-AP | 1:1000 |  |  |  |
| FZD9 | Proteintech | 13865-1-AP | 1:1000 |  |  |  |
| α-Tubulin | Abcam | ab7291 |  |  |  | 1:500 |
